# Stabilizing α-Helicity of a Polypeptide in Aqueous Urea: Dipole Orientation or Hydrogen Bonding?

**DOI:** 10.1021/acsmacrolett.3c00223

**Published:** 2023-06-15

**Authors:** Luis A. Baptista, Yani Zhao, Kurt Kremer, Debashish Mukherji, Robinson Cortes-Huerto

**Affiliations:** † Max Planck Institute for Polymer Research, Ackermannweg 10, 55128 Mainz, Germany; ‡ Quantum Matter Institute, University of British Columbia, Vancouver, British Columbia V6T 1Z4, Canada

## Abstract

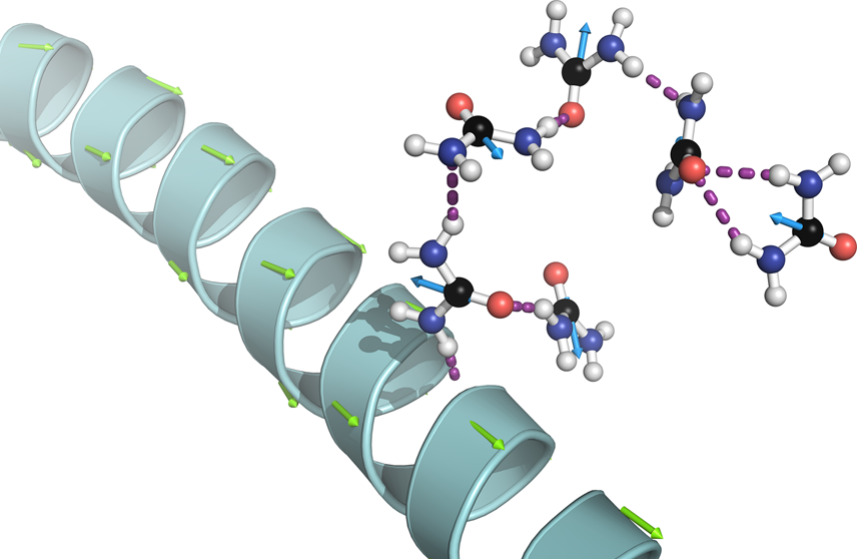

We propose a mechanism for α-helix folding of polyalanine in aqueous urea that reconciles experimental and simulation studies. Over 15 μs long, all-atom simulations reveal that, upon dehydrating the protein’s first solvation shell, a delicate balance between localized urea–residue dipole interactions and hydrogen bonds dictates polypeptide solvation properties and structure. Our work clarifies the experimentally observed tendency of these alanine-rich systems to form secondary structures at low and intermediate urea concentrations. Moreover, it is consistent with the commonly accepted hydrogen-bond-induced helix unfolding, dominant at high urea concentrations. These results establish a structure–property relationship highlighting the importance of microscopic dipole–dipole orientations/interactions for the operational understanding of macroscopic protein solvation.

Understanding (bio)macromolecular solvation in solvents, especially in binary solvents, is at the onset of many developments in designing biocompatible materials.^1−3^ Interesting examples include, but are not limited to, responsiveness of polymers and hydrogels/microgels to external stimuli,^4−6^ self-assembly of complex structures in solutions,^7,8^ and denaturation of proteins.^9−11^ In these cases, the solubility, and thus structure, of a solute in water gets severely affected by the presence of osmolytes within the solvation shell, such as small alcohols, urea, and ions. Here, especially, urea is a common osmolyte known to denature a native structure of a polypeptide sequence or a protein in general. Indeed, urea–peptide interaction is somewhat nontrivial given that many competing interactions govern the macroscopic solvation behavior.^12−14^ In this context, most studies have discussed the importance of weak van der Waals (vdW) forces, the strength of which is less than a *k*_B_*T* at room temperature *T* = 298 K, and relatively stronger hydrogen bonds (H–bond), the strength of which is between 4–8*k*_B_*T*.^5,15,16^ Here, *k*_B_ is the Boltzmann constant. The generally accepted view of the microscopic origin of protein denaturation in aqueous urea states that the urea molecules preferentially interact with the protein backbone, impacting the hydrophobic core and thus the unfolding of a sequence.^13,14^ In this context, NMR experiments of alanine-rich polypeptides have suggested that the driving force for the urea-induced denaturation is due to a high tendency to form urea–residue H-bonds^17^ that disrupts the residue–residue H-bond network responsible for stabilizing a helical conformation. Consistent with this common wisdom, circular dichroism (CD) spectroscopy results have shown that the α – helix content, quantified in terms of mean residue ellipticity, gradually decreases with increasing urea molar concentration *c*_u_ below 4.0 M.^18^ Ideally, if all residues denature in aqueous urea mixtures, one should expect a relatively rapid decrease in the α-helix content with *c*_u_. However, the observed trends in CD (i.e., a weak initial decay in residue ellipticity) might indicate that, while some residues tend to denature due to urea, specific residues may even fold under the influence of urea molecules. This observation is further supported by a recent set of experiments where it has been shown that poly-alanine shows an increased degree of stable secondary structure in aqueous urea mixtures.^19^

The CD results discussed above^18^ were well reproduced by an effective theoretical model. In particular, within the Zimm–Bragg description,^20^ a polypeptide is approximated as a chain of residues, each in either a helix or a coil state. By computing the equilibrium constant of helix formation, *s*, it is possible to estimate the theoretical mean residue ellipticity. The effect of urea can then be introduced using the linear extrapolation method (LEM), i.e., the shift in solvation (unfolding) free energy Δ*G*_s_ is a linear function of *c*_u_ with a slope *m*.^21^ This gives *s* = *s*_0_ exp(−*mc*_u_/*RT*), with *s*_0_ being the value of *s* at *c*_u_ = 0 M and *R* = 8.314 J mol^–1^ K^–1^ is the gas constant. By fitting the experimental mean residue ellipticity, a value of *m* = −0.12 kJ mol^–1^ M^–1^ (or −0.048 *k*_B_*T* M^–1^) per residue was obtained.^18^ Here, we note in passing that a substantial contribution to the total free energy comes from H-bonds. Thus, the *m*-value estimated above is expected to be significantly larger, simply because of the larger H-bond strength.^5,15,16^ Indeed, simulations have reported over an order of magnitude larger *m*-value than the LEM predictions,^22^ see also the Supporting Information, Section S5 and Figure S11. Therefore, the connection between these effective models, experiments, and the microscopic picture of peptide solvation needs to be adequately clarified.

Motivated by these observations, we investigated the structure–property relationship in polypeptide solvation to establish a correlation between microscopic interaction details and macroscopic conformations. In particular, we show how a delicate competition among dispersion, H-bonding, and local dipole–dipole interactions (DDI) controls the solvation behavior of a sequence. Here, it is essential to note that, to the best of our knowledge, simulations (to a large degree also experimental data) ignored the highly local DDI-based explanations, which are expected to play an essential and nontrivial role in the polypeptide solvation. Indeed, recent experimental work has commented on the importance of dipole orientations within the protein’s first solvation shell (FSS), and its direct correlation with the solvation behavior.^23^ For this purpose, we perform over 15 μs molecular dynamic simulations by taking a model system of polyalanine with 60 residues (Ala60) in aqueous urea mixtures. In addition to the role played by the large poly alanine sequences in promoting the self-assembly of intrinsically disordered proteins,^24^ the choice of Ala60 was motivated by its well-defined secondary structure.^25^ See the Supporting Information, Sections S1 and S2 for the simulation details.

In Figure 1, we start our discussion by commenting on the α-helix content as a function of *c*_u_. Note that, for the clarity of presentation, we also report the urea mole fraction *x*_u_. It can be appreciated that the data show a nonmonotonic functional dependence with *c*_u_. More specifically, different solvation regions are observed: (a) For *c*_u_ = 0.0 (i.e., in pure water), we observe a rather globular conformation with virtually zero α – helix content. It is well-known that water is a poor solvent for poly–alanine, with his α – helix being unstable in water at room-temperature.^26,27^ Our simulations reproduce these observations. (b) Ala60 expands upon increasing *c*_u_, and starting at *c*_u_ ≃ 2 M, the α-helix content grows up to 60%, which at *c*_u_ ≃ 4 M propagates almost to the whole Ala60 (see the simulation snapshots in Figure 1). This initial folding is directly in agreement with the experimentally observed stabilization of poly-alanine in aqueous urea.^19^ Furthermore, for *c*_u_ < 2 M, the experimentally observed weak reduction in the degree of secondary structure of an alanine-rich sequence Ac-Tyr-(Ala-Glu-Ala-Ala-Lys-Ala)_*k*_-Phe-NH2^18^ may also reveal that, while alanine residues have the tendency to fold, the other residues compete to denature, thus overall, a structure remains reasonably constant. (c) Within the range 4 M < *c*_u_ < 6 M, we observe an apparent destabilization of the α-helix content, with Ala60 exhibiting relatively large coil regions. Finally, there is a second α-helix stabilization at around *c*_u_ = 7 M. Here, we note in passing that, while the α-helix content shows a nonmonotonic variation, Δ*G*_s_ decreases monotonically with *c*_u_ for polyalanine.^22^ This difference suggests that Δ*G*_s_ and the conformational behavior are somewhat decoupled. A structure–thermodynamic decoupling was also observed when a polymer^28^ or an intrinsically disordered polypeptide^22^ collapses in a mixture of two miscible solvents. However, the microscopic origin of polymer collapse (i.e., a spherical globule) is qualitatively different from the trends (i.e., the stability of a secondary structure) observed in Figure 1.

**Figure 1:**
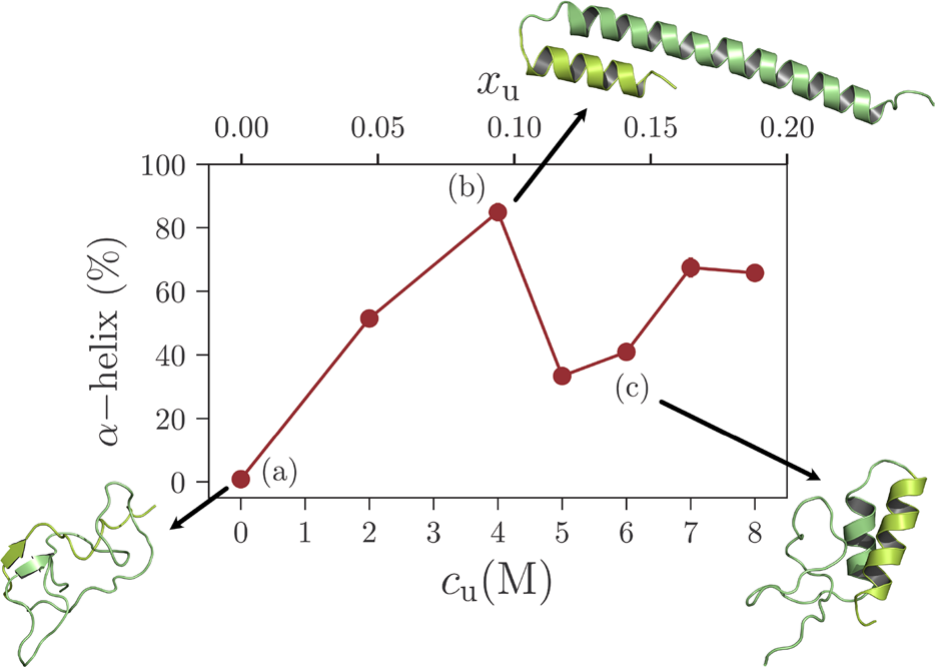
Percentage of α-helix content as a function of urea mole concentration *c*_u_. An initial monotonic increase is observed with a maximum at *c*_u_ ≃ 4 M reaching 80% of the α-helical content in the peptide. A sharp subsequent decrease in helical content between 4 M < *c*_u_ < 6 M signals at the denaturation of the protein. Representative simulations snapshots at 0, 4, and 6 M illustrate the complex conformation behavior with increasing urea concentration. Note that, for clarity of presentation, we have also included the urea mole fraction *x*_u_ in the upper panel of the abscissa. Error bars are shorter than the symbol size.

What causes such a counterintuitive structure–thermodynamic relationship? To address this issue, we will now investigate the alanine-(co)solvent coordination within the first solvation shell (FSS). To this aim, we define the cylindrical correlation functions by employing the symmetry axis indicated in the helix. See the orange arrow in Figure 2a. As expected, the data suggest an excess of urea molecules around the helix for all *c*_u_ (Supporting Information, Figure S6), indicating the well-accepted concept of preferential binding of urea with polyalanine. Note also that the generally accepted microscopic origin of this urea preferential binding to a poly peptide was shown to be dictated by the dispersion forces.^13,14^ However, if the dispersion forces were the only driving force in Ala60 solvation, Δ*G*_s_ is expected to be much weaker than 2–3 *k*_B_*T* in the concentration range 2–8 M (Supporting Information, Figure S11). Hence, other stronger interactions are also expected to be relevant. A closer inspection of the urea molecules within FSS reveals an interesting molecular arrangement. See Figure 2. It can be appreciated that the urea dipole moments (shown by the blue arrows in Figure 2a) tend to align with the dipole moment of the residue (shown by the red arrows in Figure 2a). Close to the coils, however, the urea molecules form hydrogen bonds with Ala60, represented by yellow dashed lines in Figure 2b. To quantify the degree of alignment between Ala60 and urea dipole moments, we calculate the cylindrical distribution functions (CDF) separating the parallel, antiparallel, and “middle” relative orientations (see the Supporting Information, Figure S7). Additionally, we compute the corresponding CDF for Ala60 and water (Supporting Information, Figure S8). These results indicate a preferential tendency to form antiparallel alignment between the α-helix and the urea and water dipole moments within the FSS. Within this picture, it is expected that the alignment of the dipoles corresponds to an energetically stable configuration that decreases the binding free energy of the system. Indeed, it has been revealed that molecular DDI does play an essential role in determining protein properties,^29−31^ whose precise role remains elusive.^32^ Here, a recent experimental effort has aimed to elucidate the electric dipole network within the FSS.^23^

**Figure 2:**
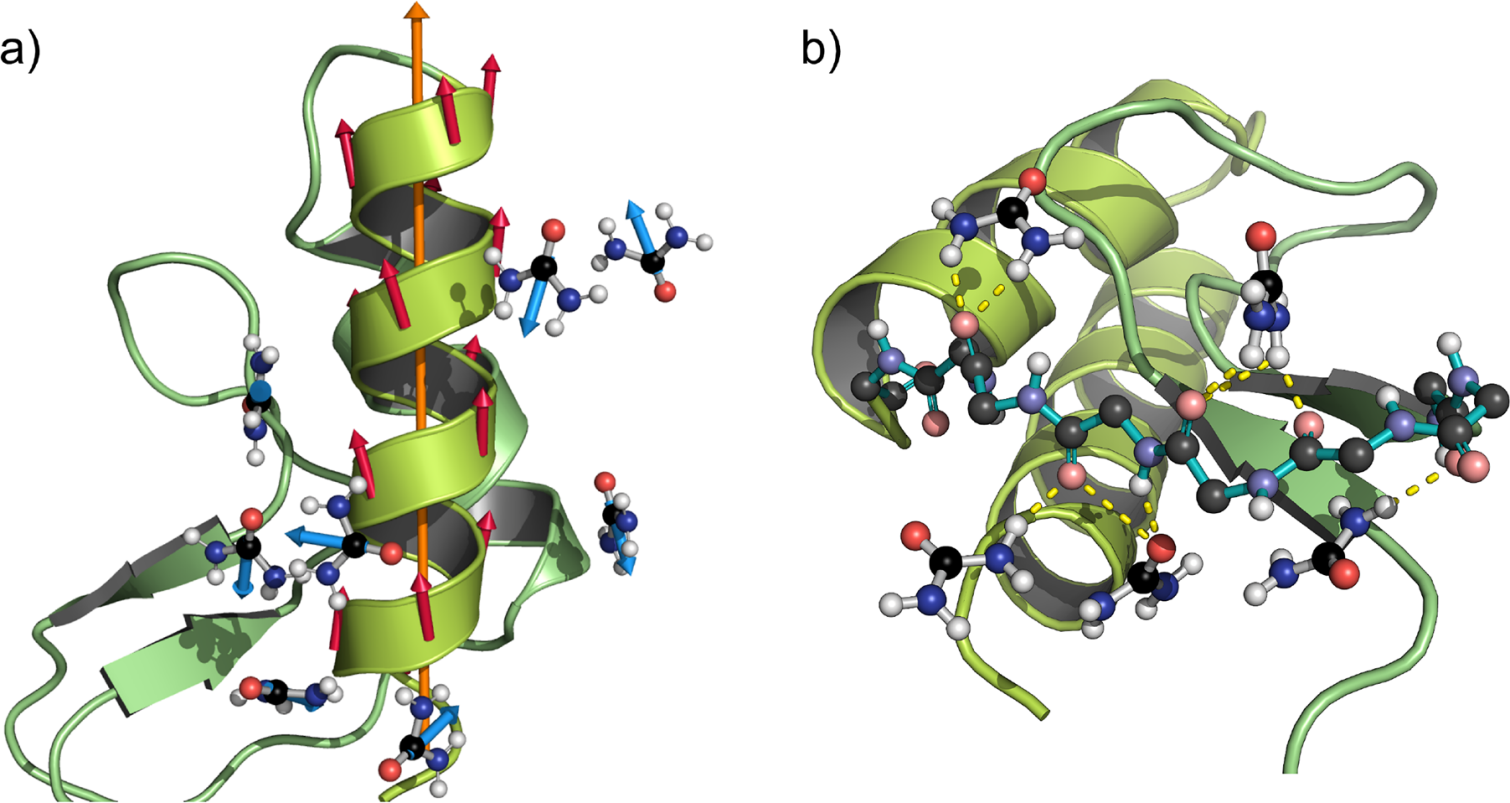
Simulation snapshots showing the detail of the orientation of urea molecules within the first solvation shell of Ala60 at 6 M. Panel (a) shows the urea molecules around the α-helix indicating urea electric dipoles (blue arrows), residue dipole (red arrows), and the total dipole of a helix (orange arrows). Panel (b) shows the urea molecules forming hydrogen bonds (yellow dashed lines) with the peptide backbone of the loop region (blue bonds). For clarity, the methyl group and hydrogen atoms are not visible in the representation.

A simple expression to estimate the energetic contribution due to DDI is the so-called Keesom energy (33) with  being the dielectric constant. Taking the average values of the dipole moments for a residue μ_r_ = 3.95 D and a urea molecule μ_u_ = 4.38 D, we find *E*_K_ ≃ −6.55 kJ mol^–1^ (or −2.64 *k*_B_*T*)^33^ for *r* = 0.6 nm (i.e., range of the first peak in the Supporting Information, Figure S6) at *T* = 298 K. This suggests that the urea–residue dipole–dipole interactions are indeed relevant. We also note in passing that *E*_K_ considers all possible orientations of the dipoles involved within *r* = 0.6 nm. In contrast, by taking the dipole moment of liquid water μ_w_ = 2.95 D separated by *r* = 0.6 nm from the residue (also from Supporting Information, Figure S6), we obtain *E*_K_ ≃ −2.97 kJ mol^–1^ (or −1.20 *k*_B_*T*). Since these water–residue DDI energy contributions are screened by thermal fluctuations, we exclude them from the discussion below. Hence, the most dominant contributions usually come from the dipole orientations between the residues within an Ala60 (termed **Scenario I**) and between a residue and the urea molecules (termed **Scenario II**). In the following, we characterize different contributions to the total resultant dipole–dipole interactions.

**Scenario I**: We first consider an α-helix, as shown by the model representation in Figure 3a,b. For this purpose, we build a perfect helix with 3.6 residues per turn and use the charge values given by the CHARMM36m force field parameters for the μ estimates.^34^ We evaluate the DDI energy on a residue *i* using *E*_*i*_ = ∑_*j*∈⟨*i*,*j*⟩_ε_*i*,*j*_,^35^ where
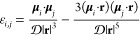
1and ⟨*i*, *j*⟩ represents the nearest neighbors of *i*, i.e., the residues *i* ± 4, *i* ± 3, *i* ± 2, and *i* ± 1. **μ**_*i*_ is the electric dipole of the *i*-th residue, and the vector **r** = **r**_*i*_ – **r**_*j*_ is the separation between dipoles *i* and *j*. Equation 1 gives *E*_*i*_ ≃ −5.29 kJ mol^–1^ (or −2.13*k*_B_*T*), indicating that the α-helix is stabilized via intramolecular DDI.

**Figure 3:**
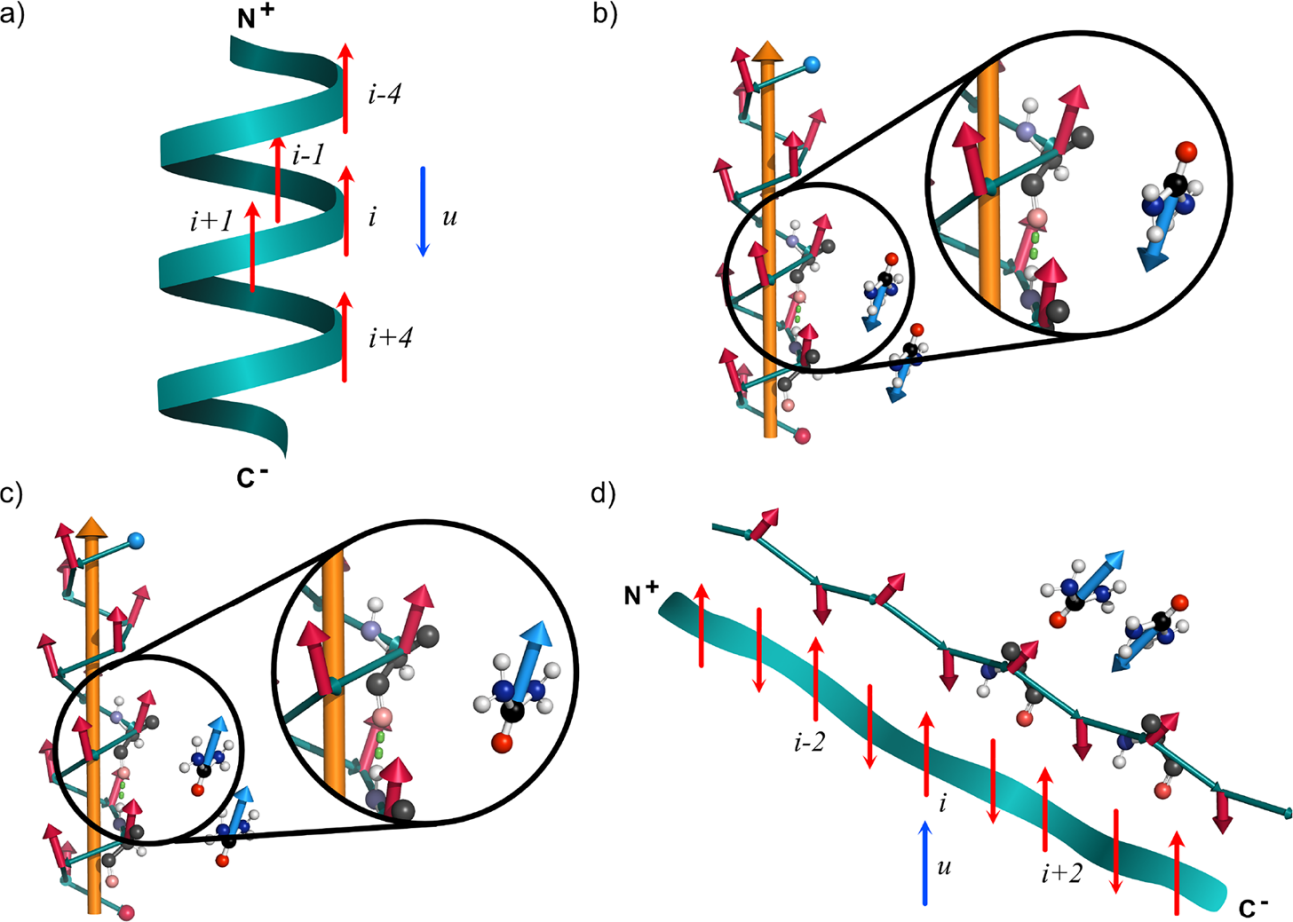
Schematic and ideal model representations of possible dipole moment configurations in the system. Panel (a) shows the α-helix conformation with the consecutive residues having the dipoles aligned (red arrows) and with neighboring urea molecules orienting their dipoles (blue arrows) side-by-side. Note that we show the most dominant relative orientation, i.e., the antiparallel arrangement of a urea molecule and the residues. Panel (b) represents the snapshot of an equivalent model system with an ideal α-helix and a urea dipole moment located at *r* = 0.6 nm. Here, an antiparallel side-by-side dipole arrangement between urea (blue arrow) and residue (red arrow) is shown. Panel (c) is the same as panel (b). However, here a urea molecule is stabilized between the *i* and *i* + 4 residues showing a parallel alignment. Panel (d) shows a fully unfolded structure, which is more stable from the point of view of dipole–dipole interactions, i.e., a local dipole-compensated arrangement. The side chain hydrogen atoms are not displayed for clarity.

**Scenario II**: (a) One possible molecular arrangement is when a urea molecule stacks side-by-side in antiparallel dipolar orientation within FSS of the residue *i*. Using the same model above, but now including the urea molecule, we obtain *E*_u_ = ∑_*j*∈⟨*u*,*j*⟩_ε_u,j_ ≃ −6.99 kJ mol^–1^ (or −2.81*k*_B_*T*) for *r* = 0.6 nm separation between the geometric centers of the residue and the urea molecule. See Figure 3b and the top panel in Supporting Information, Figure S9. (b) It is apparent from the Supporting Information, Figure S7 that urea molecules also align with a dipole moment parallel to the residue of the dipole. This arrangement is not energetically favorable for urea molecules placed side-by-side with respect to the residue. Hence, the second possible configuration is when a urea molecule sits between residues *i* and *i* + 4, for example, with dipole moments aligned in head-to-tail orientation, with the head being the NH_2_ groups of urea and the tail as carboxyl group of an alanine residue. See Figure 3c. In this case, we obtain *E*_u_ ≃ +1.89 kJ mol^–1^ (or +0.76*k*_B_*T*). Since the latter estimate is smaller than *k*_B_*T*, an by symmetry the antiparallel configuration gives *E*_u_ ≃ −1.89 kJ mol^–1^ (or −0.76*k*_B_*T*; see also the lower panel in Supporting Information, Figure S9), thermal fluctuations are expected to induce flip–flop of a urea dipole moment at this particular position and thus introduce local configurational fluctuations.

Even when the energy values discussed above suggest that the dipole interaction can stabilize an α-helix, it is still important to emphasize that DDI alone is insufficient to stabilize the secondary structure. For example, considering a test case of a fully expanded chain (shown in Figure 3d), we find *E*_*i*_ ≃ −15.95 kJ mol^–1^ (or −6.34*k*_B_*T*) and *E*_u_ ≃ −20.65 kJ mol^–1^ (or −8.20*k*_B_*T*) for an incoming urea molecule with a dipole moment aligning head-to-tail with the *i*-th residue. See Supporting Information, Figure S10. These results indicate that from the DDI point of view, the peptide unfolded state is more favorable than a stable α-helix conformation by Δ*E*_*i*_ ≃ −10.66 kJ mol^–1^ (or −4.24*k*_B_*T*). Hence, a pure dipole-based energetic argument can not fully explain the conformational behavior of an Ala60 in aqueous urea mixtures. In addition to DDI, we underline that α-helix structures are further stabilized by H-bond interactions between *i* and *i* ± 4 residues. We will return to this point in the last part of the manuscript.

Given the discussion presented above, we can now understand the nonmonotonic variation in α-helicity with *c*_u_, which is as follows:

- For *c*_u_ ≤ 4.0 M (or below 10%), i.e., when only a small amount of urea molecules is added in the aqueous solution, they preferentially arrange in the side-wise antiparallel configurations next to the Ala60 residues because of the favorable minimum energy configuration (**Scenario II** (a)) and thus dehydrate FSS of Ala60. This scenario stabilizes an α-helix structure by the urea–residue DDI, in addition to H-bond interactions between *i* and *i* ± 4 residues.
- Upon a further increase in the urea concentration, i.e., for *c*_u_ > 4 M, in addition to the side-wise arrangements discussed above, the additional urea molecules can also sit within the interstitial regions between *i* and *i* ± 4 residues (**Scenario II** (b)). In this region, and taking into account the energy considerations above, urea dipoles can flip-flop and thus disturb the intramolecular H-bond network, promoting the formation of intermolecular H-bonds between the NH_2_ groups of urea and the carboxyl groups of Ala60. These combined effects then induce the α-helix unfolding into a dipole-compensated structure within the range 4 M ≤ *c*_u_ ≤ 6 M, see Figure 1. Moreover, this urea–residue arrangement is also favorable from the viewpoint of DDI. This interpretation is consistent with experiments indicating that the urea molecules affect the intramolecular H-bond network,^17^ destabilizing the Ala60 secondary structure.
- The apparent second stability region for *c*_u_ > 6.0 M is somewhat surprising. We do not have a concrete argument for such behavior. However, based on the trajectory inspection, we speculate that the urea molecules within the FSS join hands, making a H-bonded chain-like configuration that decreases the number of urea–residue H-bonds. Indeed, urea is soluble in water at ∼9 M, thus, we expect urea molecules to aggregate at *c*_u_ > 6 M, preventing the formation of urea–residue H-bonds and constraining the DDI in the FSS. Free of urea’s strong influence, the C_α_ H-bond network reconnects, stabilizing the α-helix.

We return to the H-bond analysis to further consolidate our arguments based on the competition between DDI and H-bonds. For this purpose, we have computed the excess number of hydrogen bonds *x*_u_^exc^ = *x*_u_^H-bond^/*x*_u_ between urea and the peptide, where , with *N*_u-r_^H-bond^ and *N*_w-r_^H-bond^ being the average number of H-bonds between residue and urea and residue and water, respectively. *x*_u_^exc^ > 1 indicates an excess of H-bonds, as seen in the top panel of Figure 4. This result demonstrates that urea strongly tends to make H-bonds with Ala60, in good agreement with the NMR data.^17^ Lastly, to unravel the competition between dipole alignment and H-bond formation with *c*_u_, we define and compute excess quantities to bring together our proposed mechanism and simulation results. We focus on the average number of urea (*N*_u-r_^antiparallel^) and water (*N*_w-r_^antiparallel^) molecules in the FSS with antiparallel dipole orientation (−1 ≤ ⟨cos θ⟩ < −0.98, see the Supporting Information, Figure S7). We define the antiparallel urea mole fraction as, , which we use to compare with the fraction of H-bonds *x*_u_^H-bond^. To this end, we compute the ratio δ_H-bond_^antiparallel^ = *x*_u_^antiparallel^/*x*_u_^H-bond^ in Figure 4.

**Figure 4:**
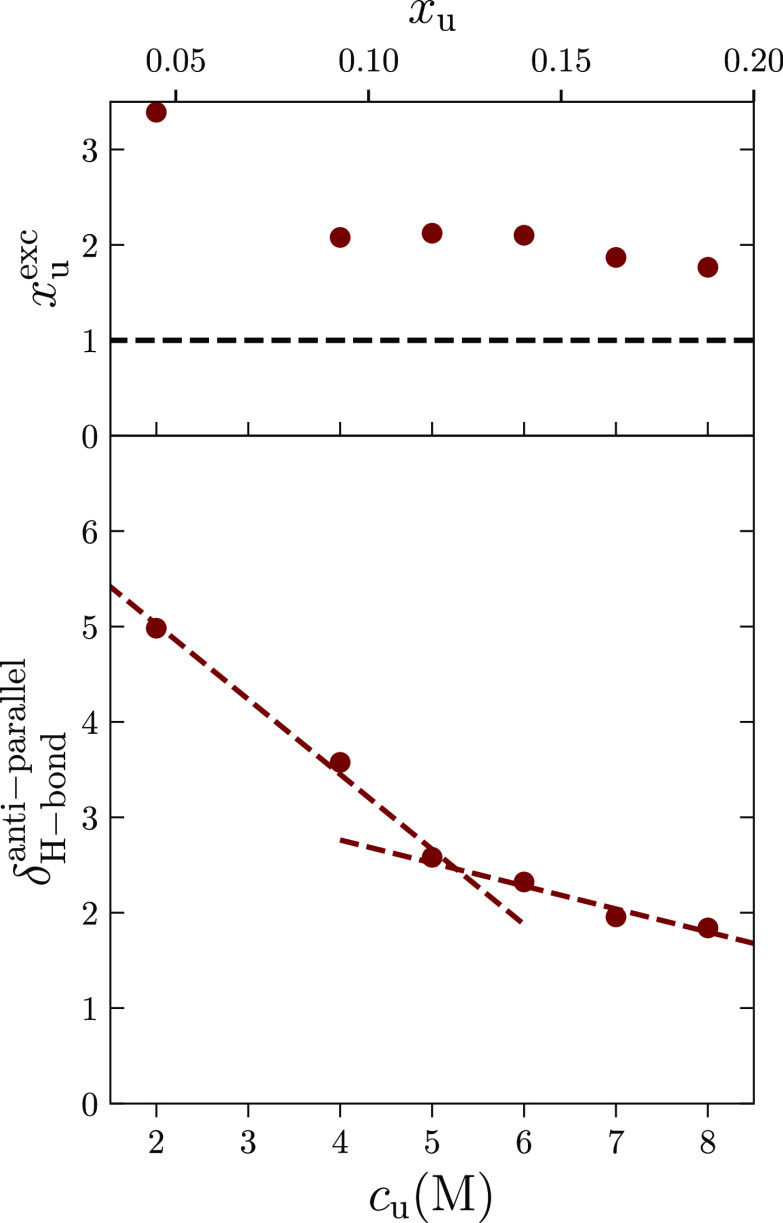
(Upper panel) Excess number of urea–peptide hydrogen bonds *x*_u_^exc^ as a function of urea mole concentration *c*_u_. The black line is a guide to the eye. The observed trend *x*_u_^exc^ > 1.0 is in agreement with the mechanism of α-helix unfolding reported experimentally^17^ and schematically depicted in Figure 3. (Lower panel) Ratio between antiparallel urea molecules in the first solvation shell and the number of urea-peptide H-bonds as a function of urea concentration. Two linear regimes with a crossover between *c*_u_ = 4 and 5 M are apparent, in registry with Figure 1. DDI dominates the solvation behavior at low concentrations, and the H-bond becomes increasingly important at high concentrations. The lines are drawn to guide the eye.

Consistent with the energy-based arguments, Figure 4 shows two linear regimes around *c*_u_ ≃ 5 M. For *c*_u_ < 5 M, the antiparallel dipole alignment is more important than H-bonds, while H-bonds dominate for *c*_u_ > 5.0 M. This trend agrees with the re-entrant behavior observed in Figure 1 that exhibits an apparent decrease in the α-helix formation at this concentration.

In conclusion, we have investigated the structure–thermodynamics relationship of polyalanine in aqueous urea mixtures using molecular dynamics simulations of an all-atom model. We emphasize the importance of a delicate competition between the H-bond and the dipole–dipole interactions that govern the macroscopic conformational behavior of the polypeptide sequence. We provide a probable microscopic picture of the observations discussed in the experimental literature.^18,19^ Our interpretation highlights that the effective models, such as the linear extrapolation method,^21^ must be adjusted to explain the polypeptide unfolding as they severely underestimate the shift in solvation free energy.^22^ This work also challenges the common understanding based solely on the preferential interaction of urea with protein via vdW forces and shows that several delicate microscopic details, such as the local dipole–dipole interactions, are responsible for the polypeptide (or proteins in general) solvation in binary mixtures. In a broad sense, our results hint at a direct route to an operational understanding of the polypeptide interactions in binary solutions. Thus, they may provide a new twist on our present understanding of protein solvation.

## Supporting Information Available

The Supporting Information contains five sections organized as follows: In Section S1, we introduce the model system and corresponding simulation details. The force field, with a particular focus on urea parameters and their validation, is presented in Section S2. Correlation functions, taking into account the geometry of the problem, are defined and presented in Section S3. The dipole interaction model used to interpret our results is presented in Section S4. Finally, we describe the calculation of the solvation free energy of Ala60 in Section S5 (PDF)
