# Supplementary material for: Stabilizing α-Helicity of a Polypeptide in Aqueous Urea: Dipole Orientation or Hydrogen Bonding?
Source: ACS Macro Lett. 2023 Jun 15;12(7):841–7. doi: 10.1021/acsmacrolett.3c00223 (PMC10357586; doi:10.1021/acsmacrolett.3c00223)
Supplement: Supplementary file 1 — mz3c00223_si_001.pdf [file mz3c00223_si_001.pdf]

Supplementary Material: "Stabilizing $\alpha-$helicity of a polypeptide in aqueous urea: Dipole orientation or hydrogen bonding?

Luis A. Baptista,[1] Yani Zhao,[1] Kurt Kremer,[1] Debashish Mukherji,[2] and Robinson Cortes-Huerto[1]

[1]*Max Planck Institute for Polymer Research, Ackermannweg 10, 55128 Mainz, Germany*

[2]*Quantum Matter Institute, University of British Columbia, Vancouver BC V6T 1Z4, Canada*

This supplementary document provides additional supporting information for some of the results presented in the main draft.

S1. SYSTEM, MODEL AND METHOD

Our system consists of a sixty–residue long poly–alanine (Ala60) solvated in aqueous urea mixtures with six different urea molar concentrations c_u, ranging between 2.0–8.0 M. Schematic representations of the molecular structures are shown in Fig. S1. The systems consist of a total number of solvent molecules $N \simeq 20,000$, i.e., including both water and urea molecules. The equilibrium box sizes for all these systems are $L \simeq 9.00$ nm, which is 5–6 times the typical correlation length in these systems and thus are large enough to neglect the finite size effects [1]. Initial configurations are generated by placing a completely unfolded Ala60 (i.e., a random coil) within the simulation domains, and then the water and the urea molecules are added randomly for a given c_u and at a density very close to the values known under the ambient conditions [2].

The simulations are performed using the GROMACS molecular dynamics package [3]. Individual systems are initially equilibrated for 25 ns in the canonical ensemble. Subsequently, the production runs are performed for 2 μs each configuration and thus these simulations cumulatively collect over 15 μs data. The observables, such as the $\alpha-$helix content and the number of intra– and inter–molecular hydrogen bonds, are calculated over the last 1 μs data of every trajectory. The later set of simulations is performed in the constant pressure p and constant temperature T ensemble NpT. Here, $T = 298$ K is imposed using a velocity rescale thermostat with a time constant $\tau_{\rm T} = 1.0$, and $p = 1.0$ bar is maintained using the Parrinello-Rahman barostat with a time constant $\tau_{\rm p} = 2.0$. The equations of motion are integrated using the leap-frog algorithm with a time step $\triangle t = 2.0$ fs. The electrostatics are treated using the particle mesh Ewald method.

Ala60 is modelled using the CHARMM36m force field parameters [4], and the TIP3P model [5, 6] is used for the water molecules. Initially, urea-water interactions were also obtained using the CHARMM36m force field. However, a quick inspection (data not shown) demonstrated that the standard CHARMM36m parameters do not properly capture the fluctuations and thus do not help maintain the solvent equilibrium within the simulation domain, as estimated by the comparison of the Kirkwood-Buff integrals [2] between our simulations and the known experiments. Here, we note in passing that maintaining the solvent equilibrium is an essential criterion to properly capture the solvation properties [1], such as the derivatives of activity coefficients and thus the solvation–free energy did not agree with experimental results. Therefore, in this study, we have chosen a different set of urea force field parameters than the CHARMM36m parameters. The details of the parameters used for urea are presented in the next section.

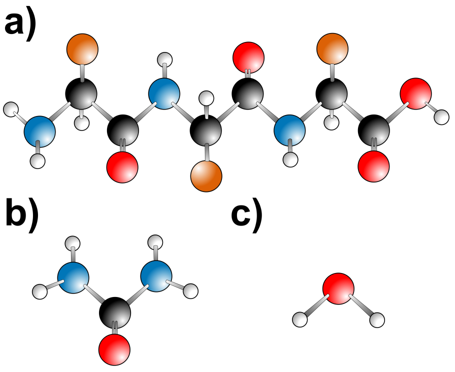

FIG. S1: Ball-and-stick representation of the chemical structures of (a) $(\mathrm{Ala})_5$, (b) urea and (c) water molecules.

S2. FORCE FIELD PARAMETERS FOR THE UREA MOLECULES

We follow the procedure described in Ref. [2] to parametrize the urea interactions. There, the force field parametrization for urea aims at reproducing the experimental Kirkwood-Buff integrals of aqueous urea mixtures [7, 8]. For a binary mixture of two species i, j in the thermodynamic limit (TL), the Kirkwood-Buff integral (KBI) is defined as,

$$G_{ij} \equiv G_{ij}^{\rm TL} = V\left[\frac{\langle N_i N_j\rangle - \langle N_i\rangle\langle N_j\rangle}{\langle N_i\rangle\langle N_j\rangle} - \frac{\delta_{ij}}{\langle N_i\rangle}\right] = 4\pi\int_0^\infty [g_{ij}^{\rm TL}(r) - 1]r^2 {\rm d}r, \tag{1}$$

where δ_{ij} is the Kronecker delta and $g_{ij}(r)$ is the multicomponent radial distribution function. Note that even the large–scale simulation setups are extremely small in

TABLE S1: The nonbonded parameters of urea molecules. $q_{\rm CHARMM}$ and $q_{\rm KBI}$ are the partial charges in the CHARMM36m and KBI derived urea models, respectively. The combination rules are $\sigma_{ij} = \frac{1}{2}(\sigma_i + \sigma_j)$ and $\epsilon_{ij} = \sqrt{\epsilon_i \epsilon_j}$.

atom	ϵ [kJ/mol]	σ [nm]	$q_{\rm CHARMM}$	$q_{\rm KBI}$ [2]
C	0.2929	0.3564	0.600	0.921
O	0.5021	0.3029	-0.580	-0.675
N	0.8368	0.3296	-0.690	-0.693
H	0.1925	0.0400	0.340	0.285

comparison to TL. Therefore, we follow the procedure described in Ref. [9] to efficiently calculate KBI from a mid–sized simulation domain.

In our case, we still use the CHARMM36m parameters for the bonded and the non-bonded interactions, but modify the partial charges on the urea molecules. Furthermore, since the bonded and the non-bonded urea-urea interactions are similar between CHARMM36m and GROMOS96, as parametrised in Ref.[2], we have taken a "reasonable" estimate by using the same partial charges reported in Ref.~[2]. We present the non-bonded parameters of the urea molecules in Table S1, where $q_{\rm CHARMM}$ and $q_{\rm KBI}$ are the default CHARMM36m and the KBI-obtained charges [2], respectively.

To test the choice of urea force field parameters, we have calculated various structural, dynamic and thermodynamic properties and compared them with the existing experimental data. In Figure S2 we compute density ρ and diffusion constants of urea $D_{\rm u}$ and water $D_{\rm w}$ as a function of urea mole concentration $c_{\rm u}$. The agreement between the experimental [2] and simulation density is quite reasonable. The simulation data of the diffusion coefficients, on the other hand, are overestimated with respect to the experimental values [2]. However, this is somewhat expected since the partial charges were modified to reproduce KBI, as is needed for our study, and not the dynamic properties of the system. Nevertheless, the simulated diffusion constants follow the experimental trend.

We have also computed the excess coordination numbers (see Figure S3), defined as $N_{ij} = \rho_j G_{ij}$ with ρ_i being the number density of species i. In this case, and as expected, the simulation results compare well with the experimental results.

Partial molar volumes, $\bar{V}_i$, and isothermal compressibilities, κ_T, can be also obtained from the KBI, [10] using

$$\bar{V}_{\rm u} = \frac{1 + \rho_{\rm w}(G_{\rm ww} - G_{\rm uw})}{\eta},$$
$$\bar{V}_{\rm w} = \frac{1 + \rho_{\rm u}(G_{\rm uu} - G_{\rm uw})}{\eta}, \textit{and}$$
$$\kappa_T = \frac{\beta\zeta}{\eta}.$$

Where $\eta = \rho_{\rm u} + \rho_{\rm w} + \rho_{\rm u}\rho_{\rm w}(G_{\rm uu} + G_{\rm ww} - 2G_{\rm uw})$, $\zeta = 1 + \rho_{\rm u}G_{\rm uu} + \rho_{\rm w}G_{\rm ww} + \rho_{\rm u}\rho_{\rm w}(G_{\rm uu}G_{\rm ww} - G_{\rm uw}^2)$ and $\beta^{-1} = k_{\rm B}T$

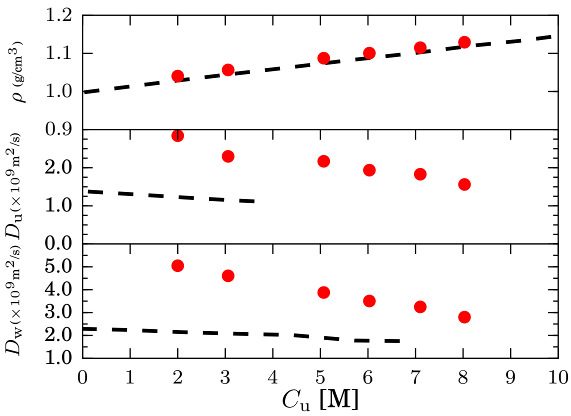

FIG. S2: The total mass density ρ and the diffusion constants of urea $D_{\rm u}$ and water $D_{\rm w}$ as a function of urea mole concentration $c_{\rm u}$. The lines correspond to the experimental data [2] and the data points are our simulation results.

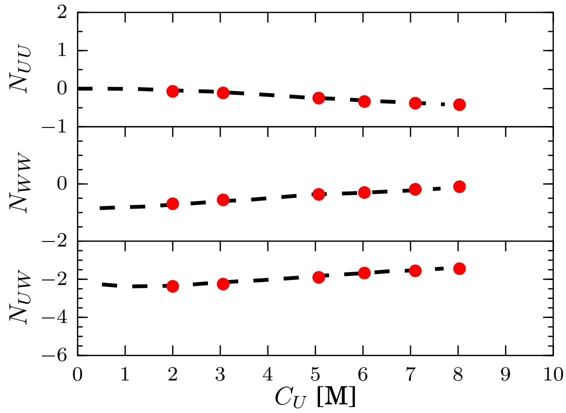

FIG. S3: Excess coordination numbers N_{ij} between solution components. The lines correspond to the experimental data [2] and the data points are our simulation results.

with $k_{\rm B}$ the Boltzmann constant. In Figure S4 we show the comparison between experimental and simulation results.

Finally, we compute the derivative of the urea molar activity, [10]

$$\gamma_{\rm uu} = 1 + \left(\frac{\partial \ln \gamma_{\rm u}}{\partial \ln \rho_{\rm u}}\right)_{P,T} = \frac{1}{1 + \rho_{\rm u}(G_{\rm uu} - G_{\rm uw})}, \quad (2)$$

with $\gamma_{\rm u}$ and $k_{\rm B}T \ln \gamma_{\rm u}$ the urea activity coefficient and chemical potential, respectively. Results for KBI-derived GROMOS96 and CHARMM36m force fields are presented in Figure S5, together with the experimental values as reported in Ref. 2. An excellent agreement between the experimental and simulation data further suggests that the choice of the partial charges could reasonably capture the thermodynamic properties.

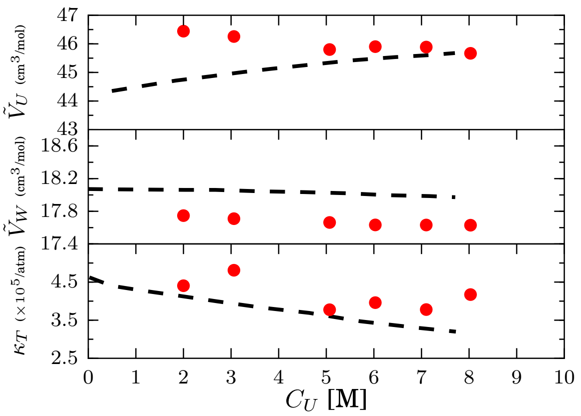

FIG. S4: Partial molar volumes $\bar{V}_i$ and isothermal compressibility κ_T. The lines correspond to the experimental data presented in Ref. 2 and the data points are our simulation results.

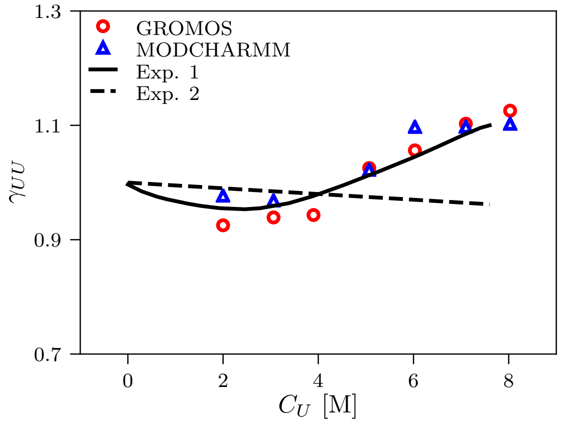

FIG. S5: Activity derivatives γ_{uu} as a function of urea mole concentration c_{u}. The lines correspond to two sets of experimental data presented in Ref. [2]. Open circles (red) are simulation results obtained with the Kirkwood-Buff derived GROMOS96 force field [2]. Triangles (blue) are the results obtained with our KB Charmm36m force field.

S3. CORRELATION FUNCTIONS

In the main text, we have discussed the results based on the coordination and orientation of the urea molecules with the first solvation shell (FSS) around the $\alpha-$helix. In this section, we provide additional data to support our claims. In this case, an $\alpha-$helix defines a local axial symmetry that we identify along the $z-$axis. With respect to this axis, we discretize the $\alpha-$helix in N_z sections of height dz, that roughly correspond to one turn (or ≈ 3.6 residues).

A. Position correlation

The cylindrical distribution function $g_i(r)$ between the geometric center of this helix segment and a representative atom of the molecule of type i using,

$$g_i(r) = \frac{1}{N_z} \sum_{k=1}^{N_z} p_i^k(r)\,, \qquad (3)$$

where

$$p_i^k(r) = \frac{\mathrm{d}n_i^k(r)}{2\pi r \mathrm{d}r \mathrm{d}z \rho_i} \qquad (4)$$

with $\mathrm{d}n_i^k(r)$ the number of i-molecules inside the k-cylindrical shell, which is located at a distance r from the helical axis, with thickness $\mathrm{d}r$ and height $\mathrm{d}z$. $g_i(r)$ for different different c_{u} are shown in Figure S6. Preferential

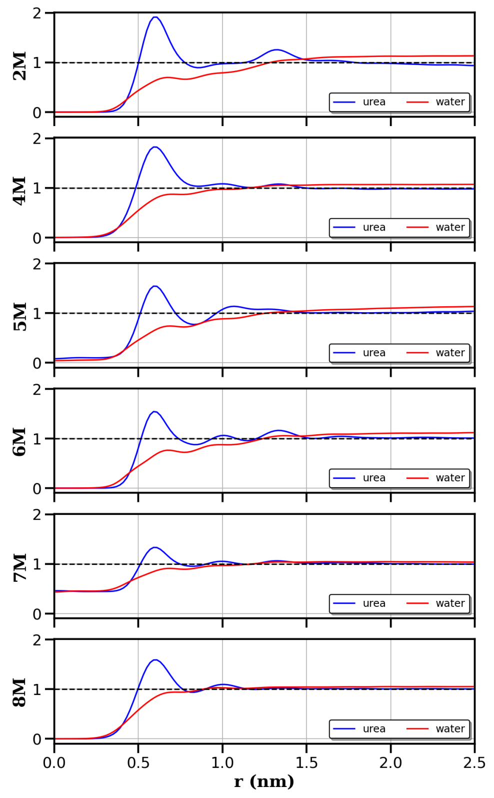

FIG. S6: Cylindrical distribution functions $g_i(r)$ calculated between the geometric centres of the residues belonging to the $\alpha-$helix and the urea (top panel) and water (bottom panel) molecules. The urea molecules tend to dehydrate the first solvation shell (FSS) of the peptide for all concentrations considered here. A non-zero contribution can be seen below 0.5 nm due to urea molecules being located at the tip of the $\alpha-$helix.

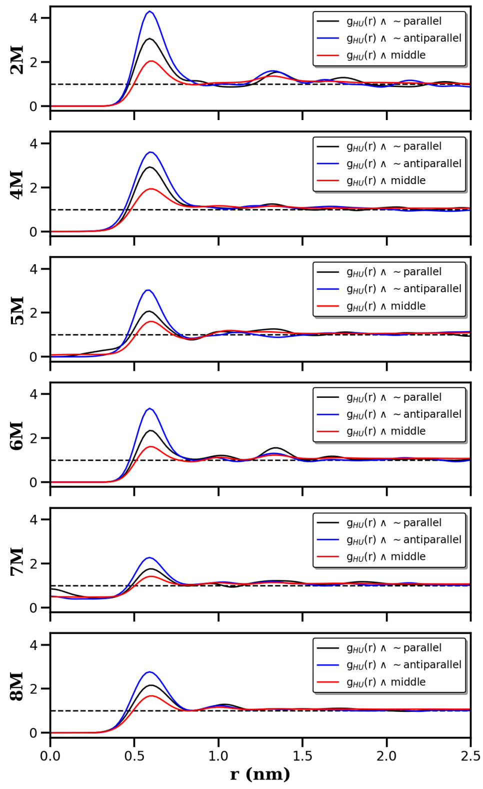

FIG. S7: Same as Figure S6 but filtered as to separate contributions from parallel ($-1 \leq \cos\theta < -0.98$, black), antiparallel ($0.98 \leq \cos\theta \leq 1$, blue) and "middle" ($-0.98 \leq \cos\theta < 0.98$, red) relative helix–urea dipole orientations.

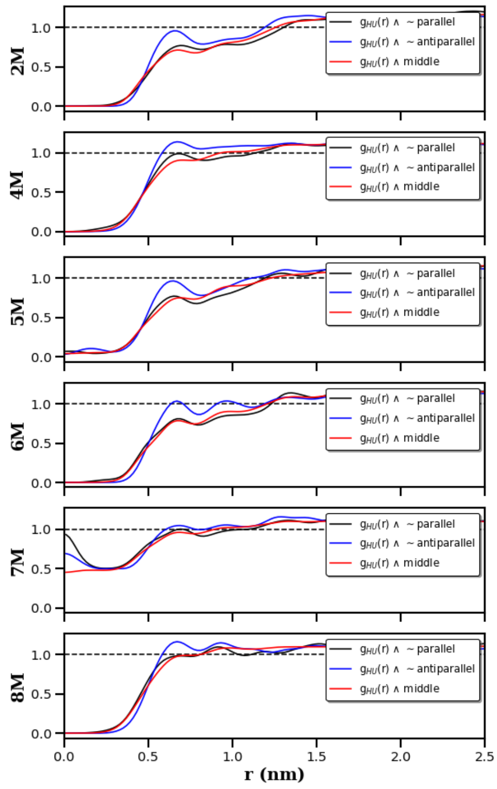

FIG. S8: Same as Figure S6 but filtered as to separate contributions from parallel ($-1 \leq \cos\theta < -0.98$, black), antiparallel ($0.98 \leq \cos\theta \leq 1$, blue) and "middle" ($-0.98 \leq \cos\theta < 0.98$, red) relative helix– water dipole orientations.

coordination of the urea molecules with the poly–alanine is clearly visible.

B. Orientation correlation

To verify whether the local dipole orientation discussed in the main text is statistically significant, we have filtered $g_i(r)$ as to separate contributions from different helix–dipole orientations: parallel ($-1 \leq \cos\theta < -0.98$), antiparallel ($0.98 \leq \cos\theta \leq 1$) and the remaining orientations ($-0.98 \leq \cos\theta < 0.98$), with θ the angle between the dipole moment of urea and the axis defined by the $\alpha-$helix. Results are presented in Figure S8. For all c_{u}, it is apparent a tendency for urea molecules in the FSS to align antiparallel and parallel with respect to the axis of the $\alpha-$helix.

S4. DIPOLE INTERACTION MODEL

To estimate the strength of the dipole-dipole interactions (DDI), we have built a model system consisting of a perfect helix with 3.6 residues per turn and use the partial charges taken from the CHARMM36m force field. [4] We use an urea molecule u as a probe and evaluate the DDI energy $E_{\mathrm{u}} = \sum_{j\in\langle \mathrm{u,j}\rangle} \varepsilon_{\mathrm{u,j}}$ as a function of the urea–residue separation r. Here, $\langle \mathrm{u,j}\rangle$ denotes the nearest neighbours (residues) of u, namely the residues $i \pm 4$, $i \pm 3$, $i \pm 2$, $i \pm 1$ and i. The DDI is estimated by [11]

$$\varepsilon_{\mathrm{u,j}} = \frac{\boldsymbol{\mu}_{\mathrm{u}} \cdot \boldsymbol{\mu}_j}{D\ |\mathbf{r}|^3} - \frac{3(\boldsymbol{\mu}_{\mathrm{u}} \cdot \mathbf{r})(\boldsymbol{\mu}_j \cdot \mathbf{r})}{D\ |\mathbf{r}|^5}\,, \tag{5}$$

with $\boldsymbol{\mu}_{\mathrm{u}}$ and $\boldsymbol{\mu}_j$ the electric dipole of the urea and j-th residue, respectively, and the vector $\mathbf{r} = \mathbf{r}_{\mathrm{u}} - \mathbf{r}_j$ being the separation between the dipoles u and j. $D = 4\pi \times 8.8541878 \times 10^{-12}$ C N^{-1}m^{-2} is the dielectric constant.

In Figure S9, we show the radial variation of E_{u} for paral-

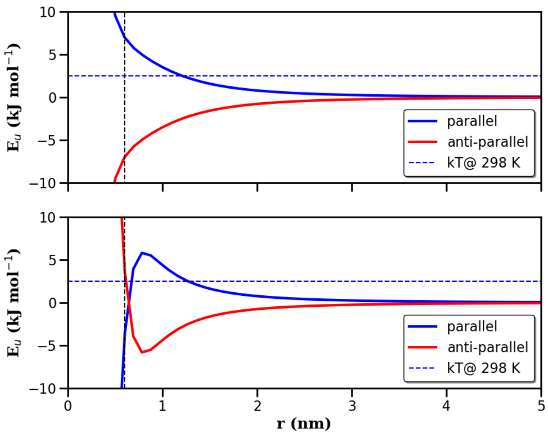

FIG. S9: Dipole-dipole potential as a function of distance r between the geometric centers of a urea molecule and the ith residue of poly-alanine. The top panel shows the data when the urea molecule makes the side–ways alignment with the residues, see the Main Figures 3a–b. The bottom panel depicts the case when a urea molecule sits between the i and $i+4$ residues, see the Main Figure 3c.

lel and anti-parallel configurations of the urea molecules from the $\alpha-$helix corresponding to the Main Figures 3a–b. Finally, in Figure S10 we show E_u for parallel and anti-parallel configurations in the unfolded structure corresponding to the Main Figure 3d.

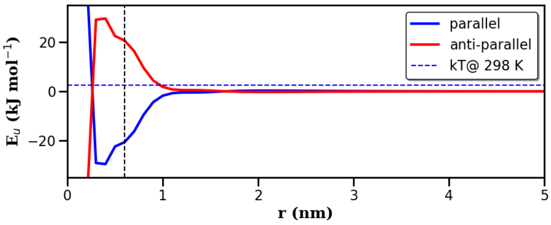

FIG. S10: Same as Figure S9, however, for a fully expanded configuration, as shown in the Main Figure 3d.

S5. SOLVATION FREE ENERGY OF POLYALANINE

When a solute, in our case an Ala60, is dissolved in a binary mixture under infinite dilution, the shift in the solvation free energy ΔG_s can be estimated within the framework of Kirkwood–Buff theory of solution via [1],

$$\lim_{\rho_\mathrm{s}\to 0}\left(\frac{\partial \Delta G_\mathrm{s}}{\partial x_\mathrm{u}}\right)_{P,T} = \frac{RT(\rho_\mathrm{w}+\rho_\mathrm{u})^2}{\eta}(G_\mathrm{pw}-G_\mathrm{pu})\,. \quad (6)$$

Here, $\rho_\mathrm{u,w}$ being the number densities of water and urea, respectively, and $\eta = \rho_\mathrm{w}+\rho_\mathrm{u}+\rho_\mathrm{w}\rho_\mathrm{u}(G_\mathrm{ww}+G_\mathrm{uu}-2G_\mathrm{wu})$. Ideally, the Kirkwood–Buff integrals G_{ij} should be calculated using Eq. 1. In the mid–sized simulation domains, however, G_{ij} are estimated by an approximate relation,

$$G_{ij} = 4\pi\int_0^R dr\, r^2\, \left[g_{ij}(r)-1\right]\,, \quad (7)$$

with $R = 2.0$ nm being the cutoff radius that is larger than the typical correlation lengths in these aqueous systems. The indices $i, j = \mathrm{p, w, u}$ labelling the peptide and water and urea molecules, respectively.

We have used the cylindrical distribution functions in Fig. S6 to evaluate G_pw and G_pu from Eq. (7). By integrating numerically Eq. (6), we obtain ΔG_s as a function of c_u, which is presented in Fig. S11. It can be appreciated that Ala60 has a preferential interaction with urea with a contrast of approximately -6 kJ/mol within the range $2 \le c_\mathrm{U} \le 8$ M.

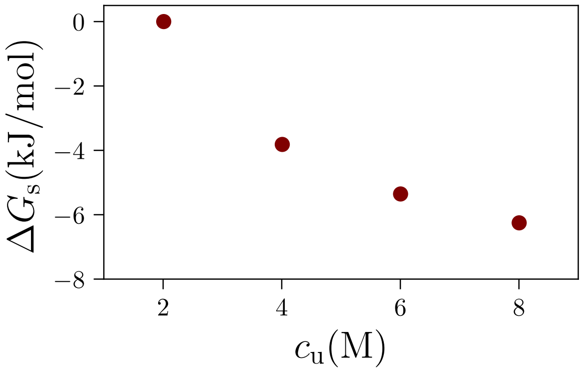

FIG. S11: Shift in solvation free energy ΔG_s per residue as a function of urea molar concentration c_u. It has been calculated from the cylindrical distribution functions in Fig. S6.

A linear fit to the data in Fig. S11 gives an estimate of the m–value, which we find to be at least -1.0 kJ mol^{-1} M^{-1} per residue. Note that this is consistent with the data reported in our earlier publication for an Ala3 peptide in aqueous urea solutions using a set of GROMOS force field parameters [12].

As is also highlighted in the introduction of our draft, the estimated m–values and ΔG_s in our simulations are over an order of magnitude larger than the m–value reported for an alanine–rich polypeptide sequence Ac-Tyr-(Ala-Glu-Ala-Ala-Lys-Ala)$_k$-Phe-NH2 in aqueous urea, i.e., $m = -0.12$ kJ mol^{-1} M^{-1} [13]. Here, however, a closer look into the microscopic atomic–level coordination reveals that there is a possibility for an alanine residue to form H–bonds with the water and the urea molecules, as also supported by our simulation data in the Main Fig. 4. Furthermore, the interaction strengths of H–bonds are between $4-8\ k_\mathrm{B}T$ (or 10–20 kJ/mol) and thus, the experimentally reported m–value seems rather

small. In this context, the difference between the experimental and simulation estimates may be due to: (1) An obvious difference in the specific sequences: while the experiment dealt with an alanine–rich sequence [13], our simulations investigate a pristine polyalanine chain. Therefore, the competing solvation structures of different residues in experiments might contribute to the difference. (2) Estimation of the m–values using the Linear Extrapolation Method [14] on the circular dichroism data is a rather non–trivial task. (3) Questions may also be raised on the choice of the force field parameters. Here, however, we wish to emphasize that we have obtained the m–value of a poly–alanine using two different parameter sets that give consistent results, see Fig. 7 in Ref. [12], and thus we believe to be in the correct regime.

[1] D. Mukherji, N. F. van der Vegt, and K. Kremer. Preferential solvation of triglycine in aqueous urea: An open boundary simulation approach. *J. Chem. Theor. Comp.*, 8(10):3536–3541, 2012.

[2] S. Weerasinghe and P. E. Smith. A kirkwood-buff derived force field for mixtures of urea and water. *J. Phys. Chem. B*, 107(16):3891–3898, 2003.

[3] Mark J. Abraham, Teemu Murtola, Roland Schulz, Szilárd Páll, Jeremy C. Smith, Berk Hess, and Erik Lindahl. Gromacs: High performance molecular simulations through multi-level parallelism from laptops to supercomputers. *SoftwareX*, 1:19–25, 2015.

[4] J. Huang, S. Rauscher, G. Nawrocki, T. Ran, M. Feig, B. L. de Groot, ..., and A. D. MacKerell Jr. Charmm36m: an improved force field for folded and intrinsically disordered proteins. *Nat. Methods*, 14(1):71, 2017.

[5] William L. Jorgensen, Jayaraman Chandrasekhar, Jeffry D. Madura, Roger W. Impey, and Michael L. Klein. Comparison of simple potential functions for simulating liquid water. *J. Chem. Phys.*, 79(2):926–935, 1983.

[6] Michael W. Mahoney and William L. Jorgensen. A five-site model for liquid water and the reproduction of the density anomaly by rigid, nonpolarizable potential functions. *J. Chem. Phys.*, 112(20):8910–8922, 2000.

[7] R. H. Stokes. *Aust. J. Chem.*, 20:2087, 1967.

[8] O. Miyakami, A. Saito, T. Matsuo, and K. Nakamura. *Biosci. Biotechnol. Biochem.*, 61:466469, 1997.

[9] R. Cortes-Huerto, K. Kremer, and R. Potestio. Communication: Kirkwood-buff integrals in the thermodynamic limit from small-sized molecular dynamics simulations. *J. Chem. Phys.*, 145:141103, 2016.

[10] A. Ben-Naim. *Molecular Theory of Solutions.* Oxford University Press, 2006.

[11] K.E. Van Holde, W.C. Johnson, C. Johnson, and P.S. Ho. *Principles of Physical Biochemistry.* Principles of Physical Biochemistry. Pearson/Prentice Hall, 2006.

[12] Y. Zhao, M. K. Singh, K. Kremer, R. Cortes-Huerto, and D. Mukherji. Why do elastin-like polypeptides possibly have different solvation behaviors in water-ethanol and water-urea mixtures? *Macromolecules*, 53(6):2101–2110, 2020.

[13] JM Scholtz, D Barrick, EJ York, JM Stewart, and RL Baldwin. Urea unfolding of peptide helices as a model for interpreting protein unfolding. *Proc Natl Acad Sci*, 92:185–189, 1995.

[14] Marcelo M. Santoro and D. W. Bolen. Unfolding free energy changes determined by the linear extrapolation method. 1. unfolding of phenylmethanesulfonyl .alpha.-chymotrypsin using different denaturants. *Biochemistry*, 27(21):8063–8068, 10 1988.
